# AKT/mTOR/BDNF pathway mediates the antidepressant-like effects of NAc-DBS in a mouse model of depression

**DOI:** 10.3389/fnbeh.2025.1662449

**Published:** 2025-10-29

**Authors:** Ranran Li, Xuhui Huang, Siwen Lv, Yongtao Liu, Ruijiao Li, Qianqian Li, Junyao Zhu, Wenjie Ren, Lujing Geng, Shuangping Ma, Yi Yu, Lei Wang, Wei Wang

**Affiliations:** ^1^School of Life Sciences and Technology, Henan Medical University, Xinxaing, China; ^2^Engineering Technology Research Center of Neurosense and Control of Henan Province, Xinxiang, China; ^3^Henan International Joint Laboratory of Neural Information Analysis and Drug Intelligent Design, Xinxiang, China; ^4^Department of Obstetrics and Gynecology, Genetics and Prenatal Diagnosis Center, First Affiliated Hospital of Zhengzhou University, Zhengzhou, China; ^5^Henan Collaborative Innovation Center of Prevention and Treatment of Mental Disorder, The Second Affiliated Hospital of Henan Medical University, Xinxiang, China; ^6^Henan Institutes of Health Central Plains, Henan Medical University, Xinxiang, China; ^7^Department of Physiology, School of Basic Medicine, Tongji Medical College, Huazhong University of Science and Technology, Wuhan, China

**Keywords:** NAc-DBS, depression, synaptic spine density, BDNF, AKT/mTOR pathway

## Abstract

Deep brain stimulation of the nucleus accumbens (NAc-DBS) has been shown to ameliorate depressive-like behaviors. However, the underlying mechanisms of action remain elusive. We aimed to investigate the impact of NAc-DBS on synaptic spine alterations in hippocampus in a depression mice model and unveil the possible signal pathway mediating such effects. The experimental protocol involved exposing adult mice to chronic unpredictable mild stress (CUMS) with or without NAc-DBS. Behavioral assessments were performed to evaluate the impact of NAc-DBS on emotional alterations. Local field potential (LFP) recordings were employed to examine the hippocampal neuronal activity in awake mice. Golgi-Cox staining was applied to quantify modifications in dendritic spine density. Additionally, hippocampal protein expression of postsynaptic density protein-95 (PSD-95), brain-derived neurotrophic factor (BDNF), and the protein kinase B (AKT)/mammalian target of rapamycin (mTOR) signaling pathway were analyzed. Results indicate that CUMS mice exhibited apparent depressive-like behaviors, concomitant with reduced hippocampal high gamma oscillation power and synaptic spine density. In addition, CUMS reduced the expression level of PSD-95 and BDNF in mice hippocampus, as well as phosphorylated AKT and mTOR protein. The study revealed that NAc-DBS could attenuate depression-like behaviors, restore high gamma oscillation power and enhance synaptic spine density, potentially by increasing BDNF protein expression level and activating AKT/mTOR signaling pathway. Furthermore, Rapamycin, a potent and specific mTOR inhibitor, was found to moderate the effects of NAc-DBS. These findings suggest that NAc-DBS could enhance synaptic spine density via AKT/mTOR/BDNF signal pathway, which may partially underline its potential antidepressant effects in CUMS induced depressive models.

## Introduction

Major depressive disorder (MDD), the most widespread mood disorder globally, presents with various incapacitating symptoms such as diminished motivation and inability to experience pleasure (Marwaha et al., 2023; Jadhav et al., 2025). Even with the availability of diverse treatments, a considerable proportion of MDD patients do not achieve sufficient symptom relief and remain at risk (Kamran et al., 2022; Kennedy, 2022). Deep brain stimulation (DBS) delivers controlled electrical impulses to precise cerebral regions through surgically implanted electrode arrays (Herrington et al., 2016). Recent studies propose that high-frequency stimulation of the nucleus accumbens (NAc-DBS) may serve as an innovative intervention for treatment-refractory depressive disorders (Song et al., 2024).

The nucleus accumbens (NAc), a key structure within the ventral striatum, is predominantly composed of medium spiny neurons, 95% of which are *γ*-aminobutyric acid (GABA)-nergic neurons (Al-Hasani et al., 2021; Xu et al., 2020). The NAc consists of two parts, the core and the shell, with the core receiving dopaminergic projections from the ventral tegmental area (VTA) and glutamatergic projections from the basolateral amygdala (BLA), prefrontal cortex, and hippocampus (Han et al., 2020; Park et al., 2019). It has long been considered to be a critical brain region for regulating rewarding behavior and a potential target for treating conditions such as obsessive-compulsive disorder and schizophrenia (Al-Hasani et al., 2015; Castro and Bruchas, 2019). This approach has undergone rigorous investigation as a therapeutic intervention for treatment-resistant depression (TRD) (Williams et al., 2018; Mayberg et al., 2005). Preclinical models and clinical trials have consistently validated both the efficacy and safety profile of DBS across multiple brain targets. Considering the NAc’s pivotal role in reward processing and its therapeutic potential, NAc-DBS has gained recognition as a viable intervention for depressive disorders (Roet et al., 2020; Krauss et al., 2020). NAc-DBS has been found to mitigate depressive behaviors in mouse models of depression, an effect potentially attributable to its activation of mesolimbic reward pathways (Zhou et al., 2022; Jakobs et al., 2019). However, the underlying mechanisms of the action remain largely unknown.

The hippocampus is one of the most important cerebral areas involved in memory and cognition (Lisman et al., 2017). Hippocampal neurogenesis disorders are a major cause of depression, and most depressed patients have a certain degree of hippocampal atrophy, which may be resulted from neurogenesis disorders (Tunc-Ozcan et al., 2019; Chen et al., 2014). Studies have revealed that high-frequency stimulation could induce long-term potentiation (LTP) of synapses between the hippocampus and the nucleus accumbens, which serves as a functional link between the two regions and the failure of LTP induction should account for the anhedonia exhibited by depressed mice (Dong et al., 2007; LeGates et al., 2018). In addition, hippocampal oscillation is closely related to synaptic plasticity, and reductions in gamma oscillation has been proposed as a biomarker for depression (He et al., 2021; Fitzgerald and Watson, 2018). BDNF, a neurotrophin, is widely distributed and extensively researched in the brains of mammals. Empirical studies have revealed that chronic stress stimulations resulted in changes in BDNF expression and may cause neuronal death, revealing the relationship between depressive-like behaviors and BDNF (Hamani et al., 2012). Aumand and Tierney found that DBS affected synaptic plasticity through the upregulation of neurotrophic signaling molecules, particularly BDNF (Tierney, 2018). Additionally, DBS has been found to activate the mTOR signaling pathway which is fundamental to synaptic proteins synthesis and synaptic plasticity (Xu et al., 2020; Abelaira et al., 2014; Sun et al., 2022). Furthermore, recent researches found that the mTOR/BDNF pathway in prefrontal cortex and hippocampus of mice is disrupted in depression and linked to impaired behavioral functions (Dong et al., 2019; Li et al., 2023). Therefore, it is plausible to speculate that NAc-DBS may achieve its antidepressant effect by enhancing BDNF levels and activating the AKT/mTOR pathway to control synaptic activity.

In the current investigation, NAc-DBS intervention significantly attenuated CUMS-induced depressive behaviors in murine subjects. We further found that NAc-DBS restored high gamma oscillation power and enhanced synaptic spine density in the hippocampus of CUMS mice, potentially through increasing BDNF protein level and activating the mTOR signaling pathway. The results could, at least, partially underline the potential antidepressant effects of NAc-DBS in CUMS induced depressive models.

## Materials and methods

### Mice

A total of 56 adult healthy C57BL/6 mice (2–4 months old) were used in this study, purchased from Henan Skobes Biotechnology Co, Ltd. The mice were housed in a controlled environment with a temperature of 22 °C ± 2 °C, a 12-h light/dark cycle, and free to food and water. The bedding was changed every 7 days. After the implantation of stimulating electrodes, each mouse was housed individually. The study protocol received ethical approval from Xinxiang Medical University’s Animal Ethics Committee (approval no. XYLL-20250316).

### Experimental designs

#### Experiment 1

To investigate the impact of CUMS for two weeks on depressive behaviors in mice, 20 mice aged 2–4 months were randomly distributed into two experimental groups, the control and CUMS group. Before CUMS procedure, mice were implanted with recording electrodes in the left ventral hippocampus region and allowed sufficient recovery time. The control group was maintained under standard conditions, while CUMS group mice were exposed to two weeks of CUMS. Then, behavioral testing and LFP recordings were performed.

#### Experiment 2

To determine whether depressive behaviors induced via CUMS that can be mitigated by NAc-DBS, 36 mice aged 2–4 months were randomly distributed into three experimental groups: the control group, DBS-off and DBS-on group. A week before the CUMS procedure, all mice were implanted with stimulating electrodes in the left NAc core and with recording electrodes in the left ventral hippocampus. After two weeks of CUMS, the DBS-on group received one week of NAc-DBS, while the DBS-off group did not receive any treatment. The control group was maintained under standard conditions. Subsequently, the mice were tested for behavior and body weight, followed by LFP recordings.

#### Experiment 3

To investigate whether the NAc-DBS can reverse the changes of synaptic spine density in hippocampus of depressive mice, we employed several complementary approaches. Quantitative reverse transcription PCR (RT-qPCR) was performed to measure the relative expression of PSD-95 mRNA, a synaptic plasticity marker, in the hippocampus. The Western blot was utilized to assess the PSD-95 protein expression in the hippocampus. Finally, Golgi-Cox staining was employed to visualize and quantify synaptic spine density’s changes in the hippocampus, comprehensively determining whether NAc-DBS can reverse these changes in depressive mice.

#### Experiment 4

To explore whether the NAc-DBS can relieve depressive behaviors in mice through enhancing BDNF protein expression and activating AKT/mTOR pathway. RT-qPCR and Western blots were applied to quantify the changes of AKT/mTOR pathway and the expression of BDNF in the hippocampus. Additionally, immunofluorescence staining was employed to assess the BDNF protein expression. Finally, the rapamycin was utilized to verify whether NAc-DBS exerts its effects by activating mTOR pathway. These approaches collectively allowed us to investigate the mechanisms underlying the antidepressant effects of NAc-DBS.

### CUMS

The chronic unpredictable mild stress (CUMS) protocol involved a combination of six short-term stimuli and four long-term stimuli over a 14-day period as previously reported (Zhou et al., 2022). The short-term stimuli included restraint for 1 h, exposure to 4 °C for 1 h, exposure to 50 °C in a pre-warmed chamber for 5 min, exposure to pepper smell for 4 h, cage shaking for 20 min, and tail pinch for 2 min. The long-term stimuli, each lasting 24 h, included water and food deprivation, cage tilting at 45°, and cage tilting without bedding. Each day, mice were exposed to a combination of two randomly selected short-term stimuli and one randomly selected long-term stimulus. The same stimulus was not applied on consecutive days to prevent habituation. In addition, each stimulus should appear with roughly equal frequency throughout the entire stress period.

### Electrode implantation surgery

Mice were anesthetized, and shaved the hair on their top of the head. They were then secured in a stereotaxic instrument. Erythromycin ointment was used to protect the eyes of mice from light damage, and the mice were kept warm. 75% alcohol was used to disinfect all experimental equipment and the mice’s scalps were disinfected with iodine. A stimulating electrode was implanted into the right NAc core (coordinates: −1.1 mm anterior, +1.45 mm lateral, and −4.65 mm ventral to bregma) and a recording electrode was implanted into the left ventral hippocampus core (coordinates: −3.6 mm anterior, +2.8 mm lateral, and −3.0 mm ventral to bregma). Four holes were drilled around the exposed skull to screw in the skull nail and fix the electrodes. The electrodes were secured with dental cement. Mice were placed back into clean cages, kept separate from each other, and provided with ample supply of water and food after surgery, after surgery. Mice were allowed at least seven days to recover from surgery before DBS.

### DBS

Starting from the 15th day after establishing the depression model, DBS was performed on the awake, freely moving mice. The stimulating electrode was connected to the stimulus isolator and waveform generator. Mice received a pulse wave current in the NAc area, with a frequency of 130 Hz, an amplitude of 100 μA and a pulse width of 60 μs for a duration of 1 h in their home cage. The same stimulation was received at the same time each day for one week (Kisely et al., 2018).

### LFP recordings

LFP was recorded using the Cerebus 64 channel signal acquisition system (Blackrock Inc., USA). Before the recording, mice were transferred to the testing room and acclimated for 30 min to ensure they were familiar with the environment. The LFP was recorded from mice in their homecage for 5 min when the signal was stable. The DBS treatment and LFP were staggered to avoid acute changes in LFP caused by DBS. Following the completion of behavioral and electrophysiological experiments, electrode localization was confirmed by histological examination of brain sections. Mice were excluded if the placement was inaccurate.

### Behavioral testing

Before behavioral testing, mice were handled daily at least seven days to familiarize them with the experimenters. Once the mice became accustomed to gentle handling, such as gentle face touching, we ensured that the experimenter’s contact did no adversely affect their behavior. All experiments were conducted at the same time each day. Prior to behavioral testing, mice were transferred to the testing room and acclimated for 30 min to ensure they were familiar with the environment.

#### Body weight

Before establishing the depression model, each mouse was weighed at 9:00 a.m. From the 12th to the 14th day after the start of modeling, each mouse was weighed at 9:00 a.m. daily, and the average over three days was calculated to minimize the impact of weight loss due to water and food deprivation rather than depression-like symptoms. From the 15th to the 21st day after starting DBS treatment, each mouse was weighed at 9:00 a.m. daily, and the average weight was calculated.

#### Sucrose preference test (SPT)

During training, mice had access to two identical bottles containing 1% sucrose for 24 h. Afterward, water was removed for 24 h while food remained available. The following day, these mice were placed in individual cages for 4 h and with access to two bottles: one containing 1% sucrose solution and the other containing water. After 2 h, the positions of the two bottles were switched to prevent positional bias. The intake of sucrose and water were recorded, and the sucrose preference rate was calculated using the following formula: Sucrose preference (%) = sucrose intake/(sucrose intake + water intake) × 100%.

#### Open field test (OFT)

The open field experimental apparatus includes an open field reaction box and a data acquisition and processing system. The box is a 50 × 50 × 50 cm cube with a white bottom and black inner walls. A digital camera is positioned 1.5 meters above the box, covering the whole open field reaction box. At the beginning, mice were placed in the center of the open field apparatus and allowed to explore freely for 2 min. Video recording and data collection were started simultaneously and lasted for 6 min. The total distance traveled by the mice, average speed and position preference were measured. After each experiment, the inner walls were wiped with 75% alcohol to prevent interference between subjects.

#### Tail suspension test (TST)

The tail suspension apparatus is composed of a tail suspension tester and a data acquisition and processing system. The tail suspension tester is a 25 × 25 × 50 cm rectangular prism with black top, bottom, and three sides. A digital camera is mounted 50 cm in front of the tester, covering its entire interior. At the beginning, mice’s tails were taped at the end, causing their head to hang down. Video recording and data collection lasted for 6 min, during which the mice’s immobility time was measured. Mice that climb up their tails were excluded from the study.

### Western blots

Mice were killed by cervical dislocation. Their brains were rapidly extracted, and the hippocampus was carefully isolated, stored at −80 °C. The hippocampus was homogenized in RIPA, and the supernatant was collected after centrifugation. Western blots was performed using 15% SDS-PAGE. Proteins were transferred to PVDF membranes, blocked with 5% skim milk for 1 h, and incubated overnight at 4 °C with primary antibodies. The next day, the membranes were incubated with secondary antibodies for 1 h at room temperature. Protein band gray values were analyzed and quantified using ImageJ software. The following antibodies were used: PSD-95 (Abcam, ab238135, 1:2000), BDNF (Abcam, ab108319, 1:1000), p-mTOR (Ser2448) (CST, 5536, 1:1000), mTOR (CST, 2983, 1:1000), p-AKT (Ser473) (Proteintech, 66,444-1-Ig, 1:2000), AKT (CST, 4691, 1:1000) and GAPDH (Proteintech, 81,640-5-RR, 1:5000).

### RT-qPCR

Mice were killed by cervical dislocation. Their brains were rapidly extracted, and the hippocampus was carefully isolated, stored at −80 °C. The hippocampus was ground in liquid nitrogen. Total RNA isolation from hippocampal tissue was performed with Trizol reagent, with subsequent reverse transcription to cDNA. RT-qPCR was performed using SYBR Green method. The 2^−∆∆Ct^ method was used to calculate the relative expression levels of each target gene. The primers used in RT-qPCR were as follows:

**Table 1:** 

Name	Primer sequence
GAPDH	Forward: 5’-GTGGACCTCATGGCCTACAT-3’
	Reverse: 5′-GGATGGAATTGTGAGGGAGA-3’
BDNF	Forward: 5’-TGAGCGTGTGTGACAGTATTAGC-3’
	Reverse: 5’-GCAGCCTTCCTTCGTGTAACC-3’
PSD-95	Forward: 5’-ATGATCTTCTCTCCGAGTTCCC-3’
	Reverse: 5’-GAGGAGACAAGTGGTAATCGC-3’
mTOR	Forward: 5’-ATTGACTTTGGGGACTGCTT-3’
	Reverse: 5’-GAGCACTTCCATCACGGT-3’
AKT	Forward: 5’-AGTCCCCACTCAACAACTTCT-3’
	Reverse: 5’-GAAGGTGCGCTCAATGACTG-3’

### Golgi-Cox staining

The FD Rapid GolgiStain Kit (FD Neuro Technologies Inc., United States) was used to measure the synaptic spine density. Mice were killed by cervical dislocation. Their brains were placed in a mixture of equal volumes of Solution A and Solution. After 6 h, the mixture of Solution A and Solution B was replaced with a fresh mixture. After 14 days, the brains were transferred to Solution C, protected from light, and stored at 4 °C for 3 days. The brains were sliced into 100 μm sections using a Leica cryostat microtome and mounted on glass slides. The sections were dehydrated with ethanol at increasing concentrations (50, 75, 95 and 100%) and cleared with xylene. Finally, the sections were sealed with neutral gum and observed under a microscope. Typical hippocampal neurons were selected and photographed. We used an Eclipse Ci-L photographic microscope to select the target area of the tissue for 1,000 × imaging, ensuring that the tissue filled the entire field of view as much as possible during imaging to maintain consistent background lighting for each photo. After imaging, we used Image-Pro Plus 6.0 analysis software, uniformly adopting micrometers as the standard unit. For each 1,000 × image, we measured the number of mushroom-shaped dendritic spines, stubby dendritic spines, filopodia-like dendritic spines, and thin dendritic spines within the 30–90 μm length range of the 2nd or 3rd dendritic branch on a complete neuron in the center of the image, recording both the measured length and the count of each type of dendritic spine within that length. Spine identification is primarily classified according to the following quantitative parameters: (1) Mushroom spine: head diameter to neck diameter ratio > 1.3, and head diameter > 0.5 μm; (2) Thin spine: spine length to maximum width ratio > 3.0, and head diameter < 0.5 μm; (3) Stubby spine: spine length to maximum width ratio < 1.5; (4) Filopodia: Length > 2 μm, with no distinct enlarged head structures observed along the entire axon. Its diameter usually remains constant and extremely slender < 0.3 μm.

### Immunofluorescence

Mice were anesthetized and perfused transcranially with 0.1 M PBS followed by 4% PFA. Their brains were removed and placed in 4% PFA at 4 °C for 24 h, then dewatered in 30% sucrose. Subsequently, the brain tissue was cut into 40 μm slices with a Leica cryostat microtome. The brain slices were taken out from the −20 °C refrigerator, rewarmed, and transferred to a culture dish containing 1 × TBS solution. The required slices were selected and moved into a 12-well plate. The slices were washed three times with 1 × TBS solution on a shaker for 15 min each. They were then blocked for 1 h in TBS++ solution (1 × TBS solution with 3% goat serum protein and 0.25% TritonX-100). The slices were incubated overnight at 4 °C (for more than 12 h) in TBS++ solution containing the corresponding primary antibody. After washing three times with 1 × TBS solution for 15 min each, the slices were blocked for 15 min in TBS++ solution. The secondary antibody, corresponding to the host of the primary antibody, was added to the TBS++ solution and incubated for 2 h. Note that all steps after adding the secondary antibody should be performed in the dark. The slices were washed three times with 1 × TBS solution, for 15 min each, then transferred to a dish containing 1 × PBS solution. Suitable slices were picked and placed on glass slides. After air-drying, an appropriate amount of anti-quenching sealing agent was added, and a cover slip was placed on top. The staining results could then be observed and photographed under a fluorescence microscope (Nikon, Japan).

### Statistical analysis

Experimental data were analyzed and graphed using GraphPad Prism software. The sample size estimation was conducted using the software G*Power, by assuming a type 2 error protection of 0.05 and a power of 0.80. Unpaired *t*-tests were used to compare two groups. Data containing more than two groups were tested by using analysis of variance (ANOVA) followed by a Tukey’s multiple comparisons test. All data are expressed as the mean ± SD. Statistical differences were considered when *p* < 0.05.

## Results

### CUMS induced depression-like behavior of mice

**Figure 1:**
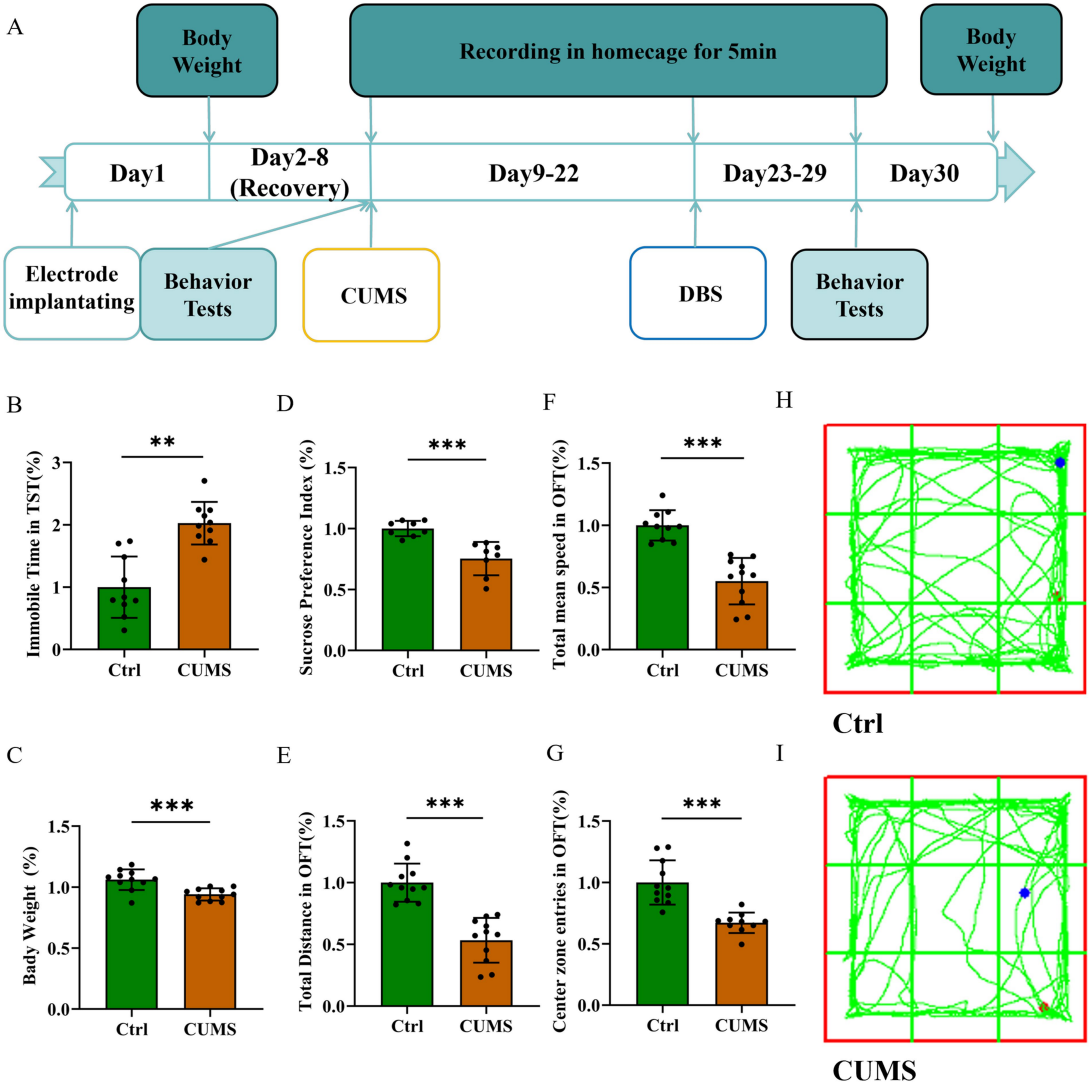
CUMS induced depression-like behavior of mice. Data are expressed as mean ± SD. **p < 0.01, ***p < 0.001. *n* = 10 per group. **(A)** Experimental timeline. **(B)** Immobile time in TST increased in the CUMS group. **(C)** The body weight decreased in the CUMS group. **(D)** Sucrose preference decreased in the CUMS group. **(E)** The total distance decreased in OFT in the CUMS group. **(F)** The total mean speed decreased in OFT in the CUMS group. **(G)** The center zone entries decreased in OFT in the CUMS group. **(H,I)** Representative trajectories of mice in OFT.

After two weeks of CUMS (Figure 1A), the CUMS group exhibited significantly increased immobility time in the TST (Figure 1B, *P* < 0.01) and weight loss (Figure 1C, *P* = 0.0006) compared to the control group. The body weight was monitored throughout the experiment as it serves as a key indicator of physical health and stress-induced metabolic alterations. Significant weight loss in mice is often associated with chronic stress exposure and may reflect reduced food intake, impaired metabolism, or energy balance dysregulation, further supporting the validity of the depression-like model (Yang et al., 2016; Jeong et al., 2013). Additionally, the CUMS group exhibited a significantly lower preference in the SPT (Figure 1D, *P* < 0.001). In the OFT, the CUMS group revealed lower total distance (Figure 1E, *P* < 0.001), slower total mean speed (Figure 1F, *P* < 0.001), and avoidance of the center (Figure 1G, *P* < 0.001). As shown in Figures 1H,I, mice in CUMS group also performed worse in the OFT. These results attested that two weeks of CUMS treatment induced the depression-like behavior in mice.

### NAc-DBS eased CUMS-induced depression-like behavior in mice

**Figure 2:**
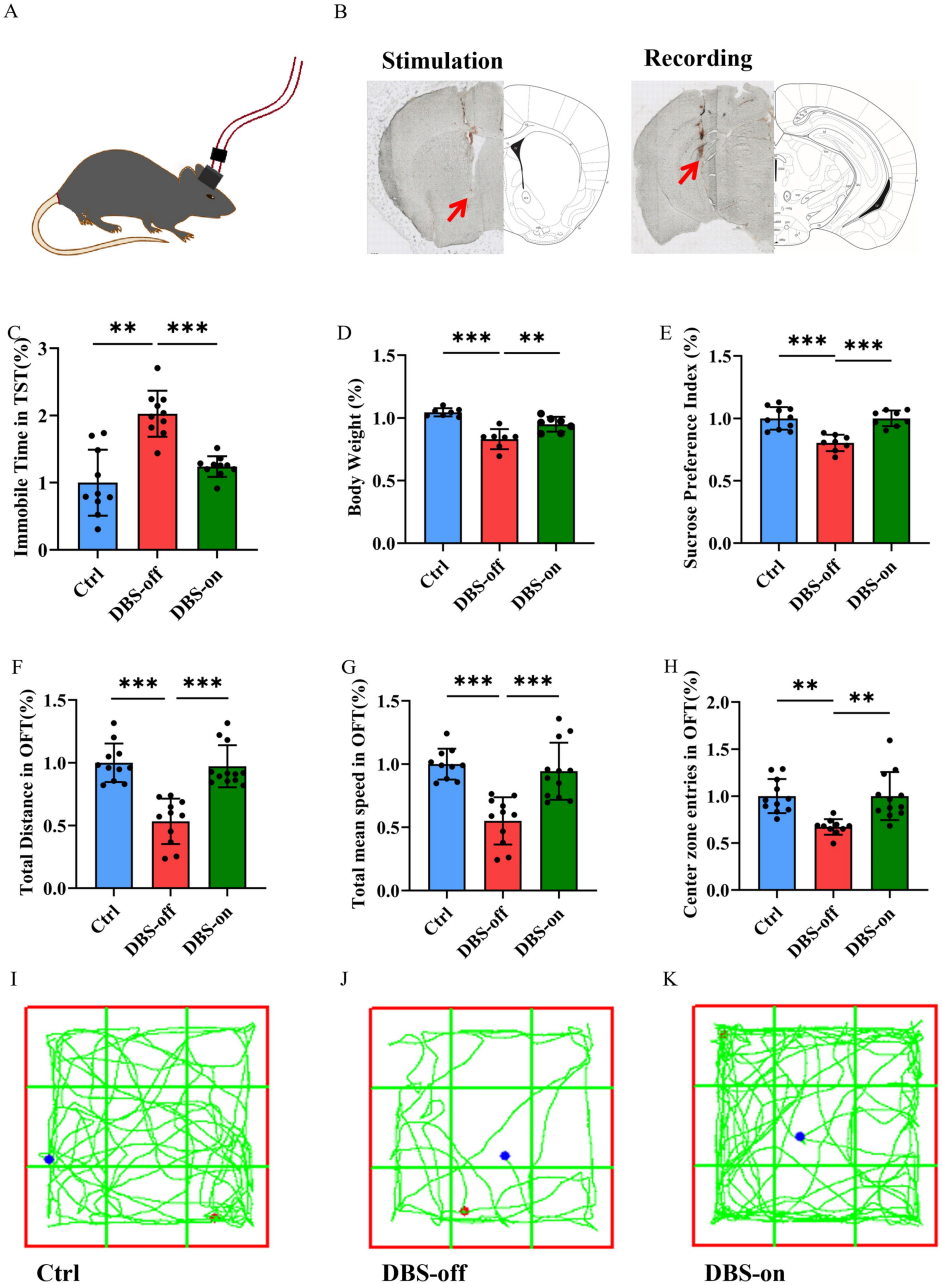
NAc-DBS reversed CUMS-induced depression-like behavior in mice. Data are expressed as mean ± SD. **p < 0.01, ***p < 0.001. **(A,B)** The stimulation electrode was implanted into the left NAc of the mouse and the recording electrode was implanted into the left vHip. Red arrows indicate the electrode position. The electrical stimulation (130 Hz, 100 μA, and 60 μs pulse width) was given 1 h per day for 7 days. **(C)** NAc-DBS reversed CUMS-induced immobile time increase in TST. *n* = 10 per group. **(D)** NAc-DBS reversed CUMS-induced body weight loss. *n* = 7 per group. **(E)** NAc-DBS reversed CUMS-induced sucrose preference decrease. *n* = 10, 8 and 8 in the control, DBS-off and DBS-on groups, respectively. **(F)** NAc-DBS reversed CUMS-induced total distance decrease in OFT. *n* = 11, 12, and 12 in the control, DBS-off, and DBS-on groups, respectively. **(G)** NAc-DBS reversed CUMS-induced total mean speed decrease in OFT. *n* = 11, 12, and 12 in the control, DBS-off, and DBS-on groups, respectively. **(H)** NAc-DBS reversed CUMS-induced center zone entries decrease in OFT. *n* = 11, 10, and 12 in the control, DBS-off, and DBS-on groups, respectively. **(I–K)** Representative trajectories of mice in OFT.

Following one week of NAc-DBS treatment for the DBS-on group (Figures 2A,B), while the DBS-off group received no treatment, the DBS-on group exhibited a decreased immobility time in TST (Figure 2C, *P* < 0.001) and showed weight gain (Figure 2D, *P* < 0.001). Conversely, the DBS-on group demonstrated a higher sucrose preference (Figure 2E, *P* < 0.001). In the OFT (Figures 2I–K), the DBS-on group showed recovery in total distance traveled (Figure 2F, *P* < 0.001), total mean speed (Figure 2G, *P* < 0.001), and center preference (Figure 2H, *P* = 0.0004). However, no significant differences were observed between the DBS-on group and the control. These results indicated that the NAc-DBS treatment could alleviate CUMS-induced depression-like behavior in mice.

### NAc-DBS recovered CUMS-induced high gamma oscillation reduction in hippocampus

**Figure 3:**
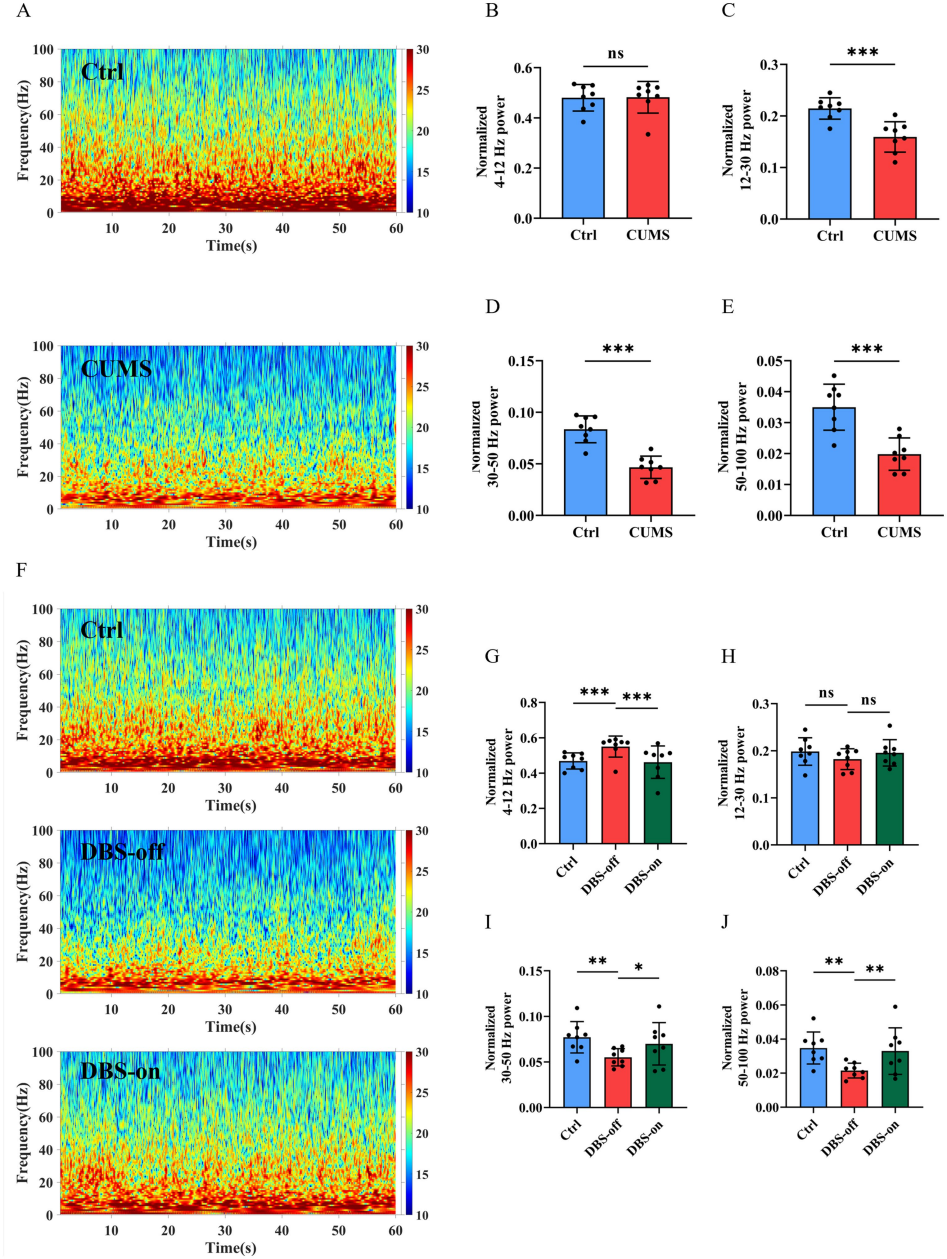
NAc-DBS reversed CUMS-induced high gamma oscillation reduction in hippocampus. Data are expressed as mean ± SD. *p < 0.05, **p < 0.01, ***p < 0.001. **(A)** Spectrograms of representative LFP in the hippocampus of mice in the home cage. Color codes indicate LFP powers of the frequency spectrum. **(B)** CUMS did not impact the Theta oscillation, *n* = 8 per group. **(C)** CUMS decreased the Beta oscillation, *n* = 8 per group. **(D)** CUMS decreased the Low gamma oscillation, *n* = 8 per group. **(E)** CUMS decreased the High gamma oscillation, *n* = 8 per group. **(F)** After NAc-DBS treatment, spectrograms of representative LFP in the hippocampus of mice in the home cage. **(G–I)** NAc-DBS caused the neuronal oscillation changes in hippocampus in the home cage (*n* = 8, 8 and 8 in the control, DBS-off and DBS-on groups, respectively). Theta oscillation **(G)**, Beta oscillation **(H)**, Low gamma oscillation **(I)**. **(J)** NAc-DBS reversed CUMS-induced High gamma oscillation decrease in hippocampus in the home cage.

To evaluate whether CUMS could cause hippocampus malfunction, we recorded the hippocampal LFP in the home cage (Figure 2B). We classified neural oscillations according to frequency into: Theta oscillation (4–12 Hz), Beta oscillation (12–30 Hz), Low Gamma oscillation (30–50 Hz), and High Gamma oscillation (50–100 Hz). After CUMS treatment, the LFP power of high gamma band was reduced after CUMS treatment (Figures 3A,E, *P* = 0.0003), while there was no significant change in the theta band (Figure 3B, *P* = 0.9415). Additionally, the beta (Figure 3C, *P* = 0.0007) and low gamma (Figure 3D, *P* < 0.001) bands showed varying degrees of reduction. Following NAc-DBS treatment, the LFP power of high gamma band in DBS-on group was restored compared to the DBS-off group (Figures 3F,J, *P* = 0.0290). Interestingly, the power of theta (Figure 3G, *P* = 0.0305) and beta (Figure 3H, *P* = 0.4392) bands showed an opposite trend. Moreover, the low gamma (Figure 3I, *P* = 0.0379) band was also restored. These results demonstrate that the LFP power of high gamma band in mice hippocampus is associated with CUMS-induced depression-like behavior and it can be restored by NAc-DBS treatment.

### NAc-DBS reversed the changes of synaptic spine density in hippocampus of CUMS mice

**Figure 4:**
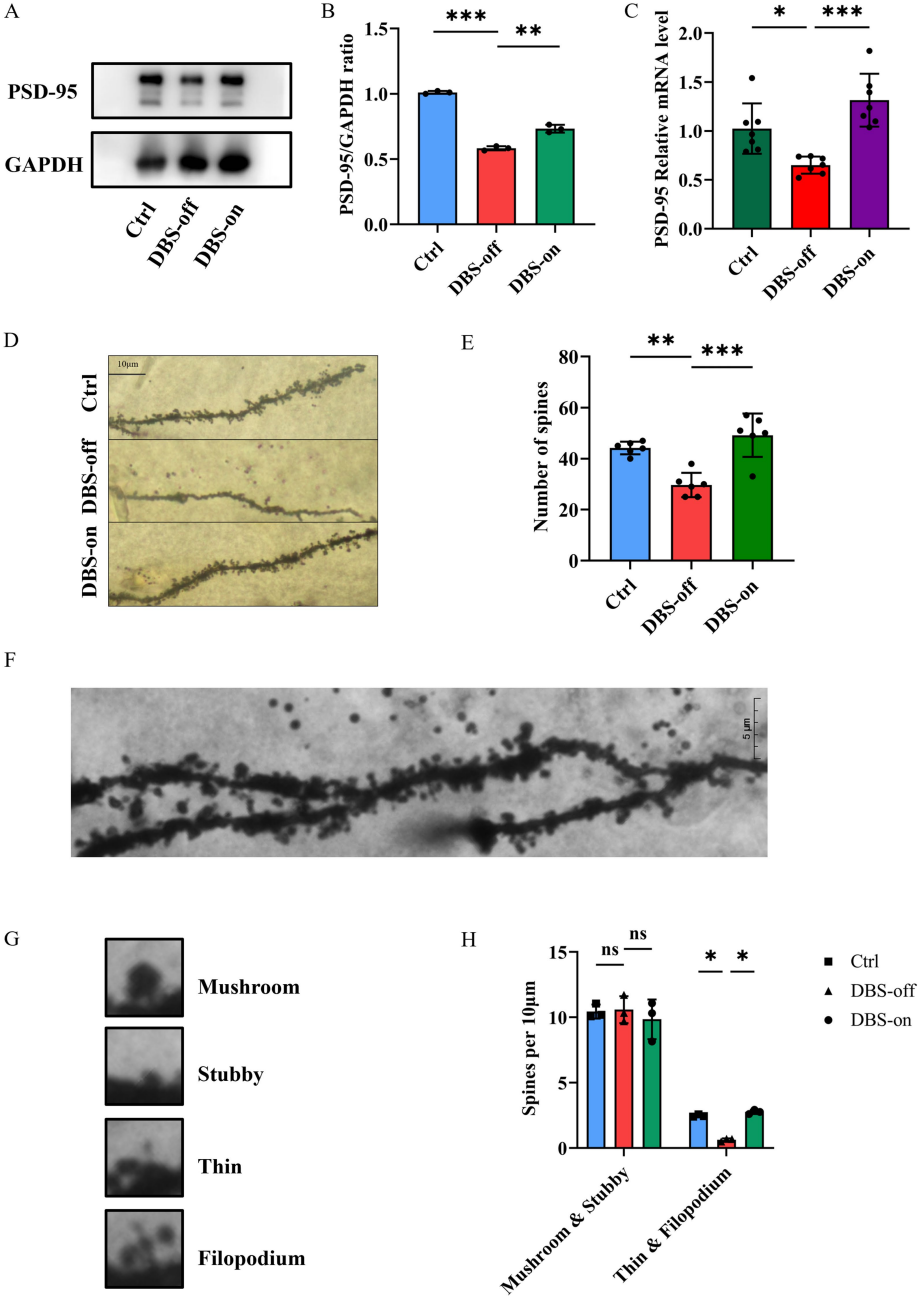
NAc-DBS reversed the changes of synaptic plasticity in hippocampus of CUMS mice. Data are expressed as mean ± SD. *p < 0.05, **p < 0.01, ***p < 0.001. *n* = 3–6 per group. **(A)** Western blots showed the expression of PSD-95 in the hippocampus from the control, DBS-off and DBS-on group. **(B)** Quantitative analysis of protein level of PSD-95 in the hippocampus. NAc-DBS reversed CUMS-induced the protein level of PSD-95 decrease. **(C)** NAc-DBS reversed CUMS-induced the gene level of PSD-95 decrease. **(D)** Representative Golgi-Cox staining images showed the synaptic spine densities on CA1 pyramidal neurons in hippocampus from the control, DBS-off and DBS-on groups. Scale bar, 10 μm. **(E)** NAc-DBS reversed CUMS-induced synaptic spine densities decrease on the CA1 pyramidal neurons. **(F)** Representative Golgi-Cox staining image of the CA1 synaptic spine under 1,000x magnification. Scale bar, 5 μm. **(G)** Images of the four different spine subtypes. **(H)** Quantification of mature and immature synaptic spine subtypes of CA1 neurons.

The PSD-95 is an key scaffolding protein on the postsynaptic membrane of excitatory neurons, which is closely linked to synaptic plasticity (Levy et al., 2015; Xu, 2011). We found that PSD-95 protein expression in the hippocampus of CUMS group mice was reduced compared to the controls. However, NAc-DBS reversed this phenomenon (Figures 4A,B, *P* < 0.001). RT-qPCR results mirrored those of Western blots (Figure 4C, *P* = 0.0004). Golgi staining showed that the number of spines in the CA1 (Figures 4D,E, *P* < 0.001) region of the hippocampus decreased in DBS-off group mice but increased in DBS-on group mice. Furthermore, no density difference was found between mature spines (mushroom and stubby), but immature spines (thin and filopodium) showed a significant increase in DBS-off mice and decrease in DBS-on mice (Figures 4F–H, *P* = 0.0209). These results suggested that NAc-DBS reverse the decrease of synaptic spine density in hippocampus of CUMS mice.

### NAc-DBS alleviated depression-like behavior in mice by enhancing the BDNF protein and activating the AKT/mTOR pathway

**Figure 5:**
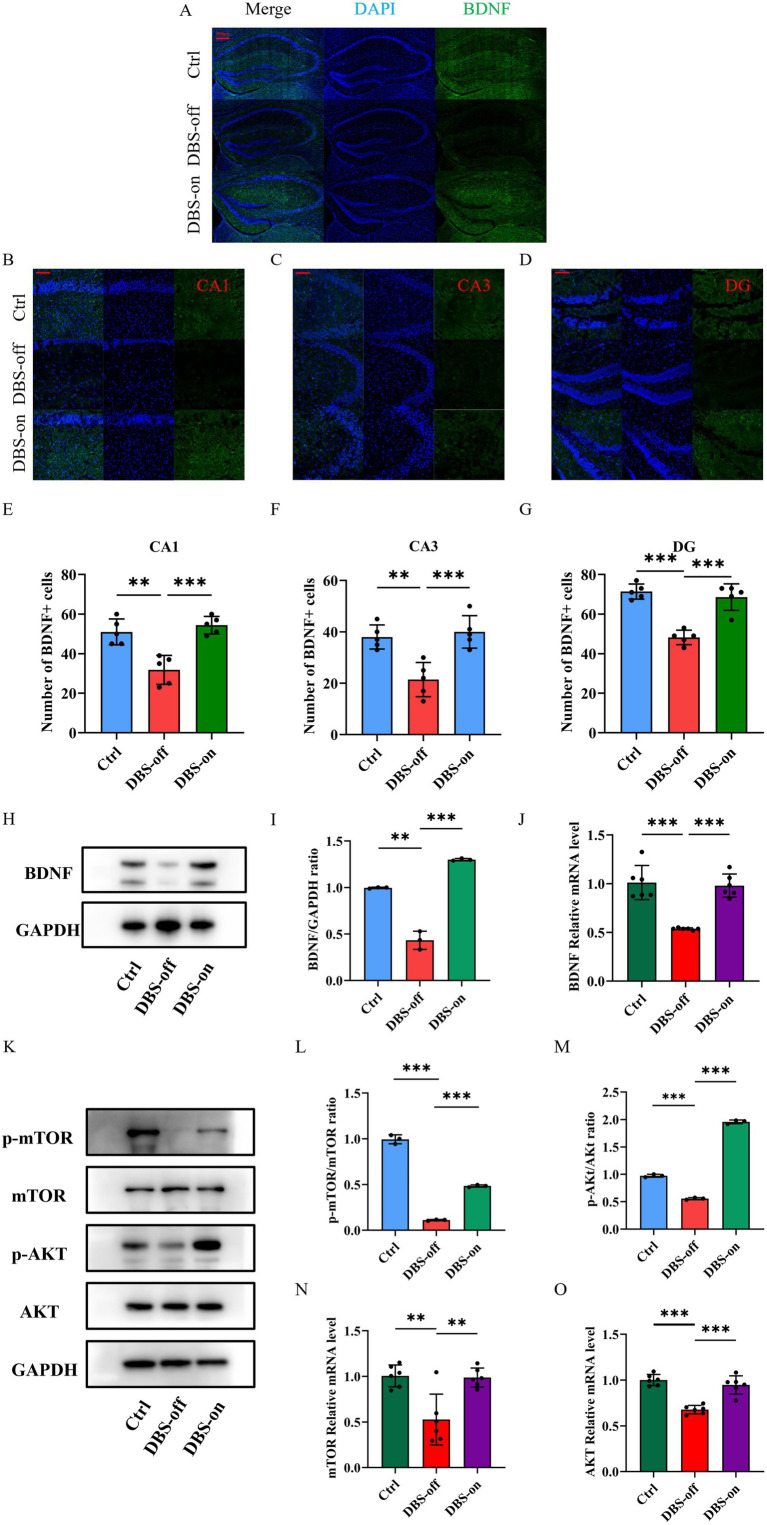
NAc-DBS reversed depression-like behavior in mice by enhancing the BDNF protein and activating the AKT/mTOR pathway. Data are expressed as mean ± SD. **p < 0.01, ***p < 0.001. *n* = 3–6 per group. **(A)** Representative Immunofluorescence staining images showing the BDNF-positive cells in the hippocampus from the control, DBS-off and DBS-on groups. Scale bar, 500 μm. **(B–D)** Representative Immunofluorescence staining images showing the BDNF-positive cells in the CA1, CA3 and DG region in hippocampus from the control, DBS-off and DBS-on groups. Scale bar, 100 μm. **(E)** NAc-DBS reversed CUMS-induced BDNF-positive cells decrease in the CA1. **(F)** NAc-DBS reversed CUMS-induced BDNF-positive cells decrease in the CA3. **(G)** NAc-DBS reversed CUMS-induced BDNF-positive cells decrease in the DG. **(H)** Western blots showed the expression of BDNF in the hippocampus from the control, DBS-off and DBS-on group. **(I)** Quantitative analysis of protein level of BDNF in the hippocampus. NAc-DBS reversed CUMS-induced the protein level of BDNF decrease. **(J)** NAc-DBS reversed CUMS-induced the gene level of PSD-95 decrease. **(K)** Western blots showed the expression of mTOR, p-mTOR, AKT and p-AKT in the hippocampus from the control, DBS-off and DBS-on group. **(L,M)** Quantitative analysis of protein level of p-mTOR/mTOR and p-AKT/AKT ratio in the hippocampus. NAc-DBS reversed CUMS-induced the protein level of p-mTOR and p-AKT ratio decrease. **(N,O)** NAc-DBS reversed CUMS-induced the gene level of mTOR and AKT decrease.

To investigate whether NAc-DBS relieves the hippocampal injuries and mitigates depression-like behavior in mice by enhancing the BDNF protein and activating the mTOR pathway, we used Western blots and RT-qPCR to detect changes in mTOR pathway. Western blots, RT-qPCR and immunofluorescence were employed to assess BDNF expression in mice hippocampus. Results showed that the gene levels of BDNF protein and mTOR pathway decreased in the hippocampus of DBS-off group mice compared with the control group and the protein levels of BDNF and p-mTOR/mTOR ratio were also decreased. Although NAc-DBS did not alter the total protein expression of the mTOR pathway in the hippocampal tissues of DBS-on mice, BDNF and mTOR mRNA levels (Figure 5J, *P* < 0.001) for mTOR and the protein expression of BDNF (Figures 5H,I, *P* < 0.001) and p-mTOR (Figures 5K,L, *P* < 0.001) were increased by NAc-DBS. Immunofluorescence results in the CA1 (Figures 5B,E, *P* < 0.001), CA3 (Figures 5C,F, *P* < 0.001), and DG (Figures 5D,G, *P* < 0.001) regions of the hippocampus (Figure 5A) were consistent with these findings. Moreover, no significant differences were observed between DBS-on mice and control mice. BDNF acts as a precursor signal for AKT, and enhancing BDNF expression activates the AKT signaling pathway. We also detected AKT expression at the protein (Figures 5K,M, *P* < 0.001) and gene levels (Figure 5O, *P* < 0.001), with results similar to those for mTOR. These results indicate that NAc-DBS alleviates CUMS-induced depression-like behavior in mice by enhancing BDNF protein and activating the AKT/mTOR pathway.

### Rapamycin depressed the antidepressant effect of NAc-DBS

**Figure 6:**
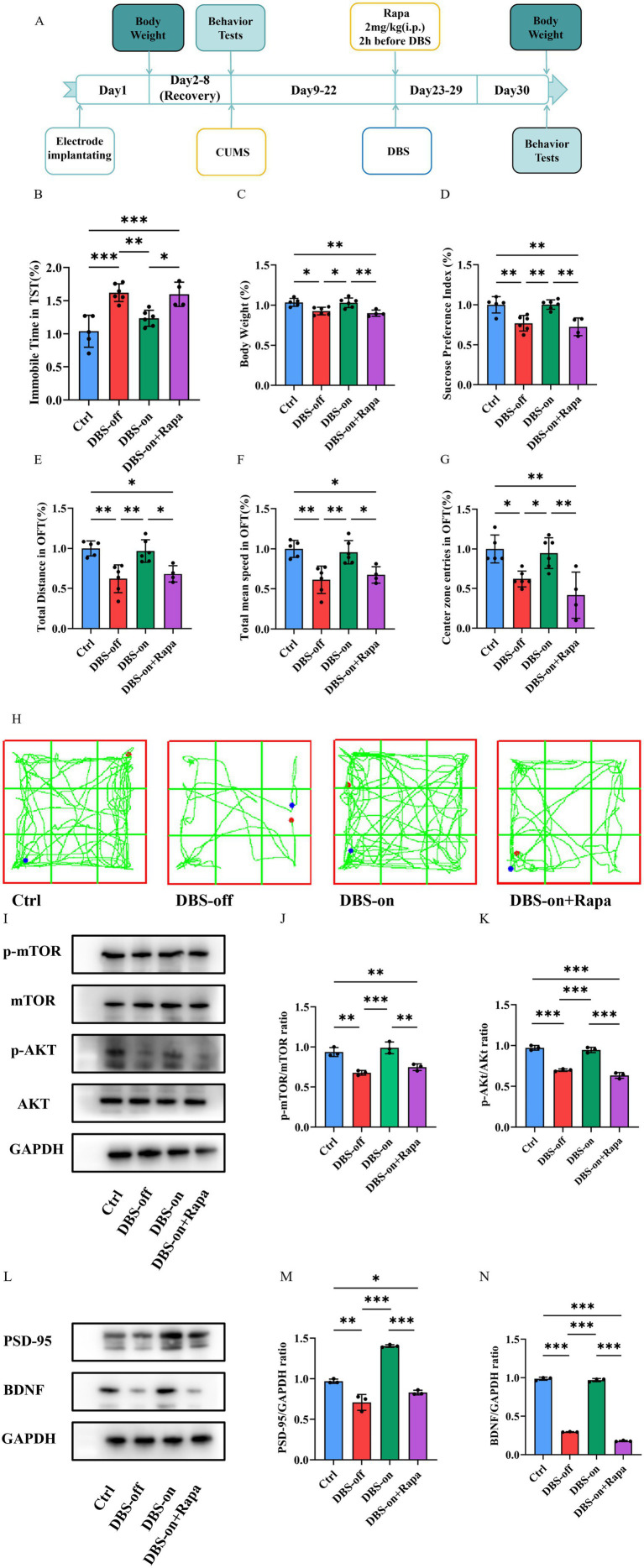
Rapamycin depressed the antidepressant effect of NAc-DBS. Data are expressed as mean ± SD. *p < 0.05, **p < 0.01, ***p < 0.001. **(A)** Experimental timeline. **(B)** Rapamycin increased the immobile time of mice in the TST which had been decreased by NAc-DBS previously. *n* = 6 per group. **(C)** Rapamycin aggravated the body weight loss which had been reversed by NAc-DBS previously. *n* = 6 per group. **(D)** Rapamycin increased the sucrose preference decrease which had been reversed by NAc-DBS previously. *n* = 6 per group. **(E)** Rapamycin decreased the total distance in the OFT which had been reversed by NAc-DBS previously. *n* = 6 per group. **(F)** Rapamycin decreased the total mean speed in the OFT which had been reversed by NAc-DBS previously. *n* = 6 per group. **(G)** Rapamycin decreased the center zone entries in the OFT which had been reversed by NAc-DBS previously. *n* = 6 per group. **(H)** Representative trajectory of mice in OFT. **(I)** Western blots showed the expression of p-mTOR/mTOR and p-AKT/AKT in the hippocampus from the control, DBS-off, DBS-on and Rapamycin group. **(J)** Quantitative analysis of protein level of p-mTOR/mTOR ratio in the hippocampus. *n* = 3 per group. **(K)** Quantitative analysis of protein level of p-AKT/AKT ratio in the hippocampus. *n* = 3 per group. **(L)** Western blots showed the expression of PSD-95 and BDNF in the hippocampus from the control, DBS-off, DBS-on and Rapamycin group. **(M)** Quantitative analysis of protein level of PSD-95/GAPDH ratio in the hippocampus. *n* = 3 per group. **(N)** Quantitative analysis of protein level of BDNF/GAPDH ratio in the hippocampus. *n* = 3 per group.

To further determine whether NAc-DBS take antidepressant effect through the mTOR pathway, we used rapamycin, a potent and specific mTOR inhibitor. Mice in the rapamycin group were administered an intraperitoneal (i.p.) injection of 2 mg/kg rapamycin 2 h before NAc-DBS treatment every day (Figure 6A) (Xu et al., 2020). In the TST, the immobility time of the rapamycin group was significantly longer than that of the control and DBS-on groups (Figure 6B, *P* < 0.001). The body weight and sucrose preference of the rapamycin group were significantly lower than those of the control and DBS-on group (Figure 6C, *P* = 0.0006; Figure 6D, *P* = 0.0001). Additionally, the total distance (Figure 6E, *P* = 0.0003), total mean speed (Figure 6F, *P* = 0.0004), and the center preference (Figure 6G, *P* = 0.0005) in the OFT were significantly affected in the rapamycin group compared to the control and DBS-on groups. As shown in Figure 6H, mice in the rapamycin group performed a worse performance in the OFT than those in control and DBS-on group. We then detected the PSD-95/BDNF protein and AKT/mTOR pathway. The results showed that the AKT/mTOR pathway which activated by NAc-DBS was inhibited by the rapamycin (Figures 6I–K, (J) *p* = 0.002; (K) *p* < 0.001). Furthermore, the expression of PSD-95/BDNF protein, which was reversed by NAc-DBS, was inhibited by rapamycin (Figures 6L–N, (M) *p* < 0.01; (N) *p* < 0.001). These results suggest that NAc-DBS alleviates CUMS-induced depression-like behavior in mice by activating the mTOR pathway.

## Discussion

Previous studies have shown that DBS could reduce the depressive behaviors in mice (Ramasubbu et al., 2020; Hamani et al., 2014; Hamani and Nóbrega, 2010). This study provides further evidence that NAc-DBS alleviates hippocampal synaptic injuries in a depressive mouse model. Specifically, we found that NAc-DBS treatment restored the reduced expression levels of both BDNF and PSD-95, as well as phosphorylated AKT and mTOR protein in the mouse hippocampus. These findings suggest that the antidepressant effect of NAc-DBS is mediated by enhancing BDNF protein expression, which in turn activates the AKT/mTOR signaling pathway, leading to the relief of CUMS-induced depression-like behaviors and hippocampal synaptic injuries in mice.

To investigate the antidepressant effect of NAc-DBS, we established a CUMS-induced depressive mouse model to observe the change of depressive behaviors in mice (Antoniuk et al., 2019). The CUMS animal model is characterized by mild stimulation, strong reliability, high effectiveness, high conformity with human depression, making it a classic animal model (Markov and Novosadova, 2022; Li et al., 2021). In our study, the CUMS induced significant depressive behaviors, including reduced sucrose preference (anhedonia) (Otte et al., 2016), weight loss, and worse performance in TST and OFT. However, the relationship between depression and weight is bidirectional and heterogeneous. Some individuals exhibit loss of appetite and weight loss (more characteristic of typical depressive symptoms), while others may display increased appetite, carbohydrate cravings, and weight gain (commonly associated with atypical depression or seasonal affective disorder). These variations can be attributed to different types of depression, neuroendocrine factors (such as hypothalamic–pituitary–adrenal axis dysfunction), medical treatment history, and unique individual stress response patterns. Therefore, simplifying the connection between depression and weight changes to a singular weight loss process represents an oversimplified interpretation of this complex clinical phenomenon. In our study, we observed a trend of weight gain accompanied by improvement in depression-like behavior following NAc-DBS treatment. We believe that NAc-DBS may effectively regulate the negative emotional state of depression. Its therapeutic effect may be achieved by restoring the normal function of the reward system and regulating normal feeding in model mice, thereby normalizing body weight while improving mood (Konttinen et al., 2019; Park et al., 2025). These effects were reversed by one-week NAc-DBS treatment, indicating that NAc-DBS alleviates the despair and anhedonia associated with depression, findings that are supported by other studies (Song et al., 2024; Shi et al., 2023; Albaugh et al., 2016).

Cognitive impairment is an important symptom of depression (Zacková et al., 2021; Culpepper et al., 2017), and changes in hippocampal synaptic plasticity may mediate this impairment (Price and Duman, 2019; Wang et al., 2019). Numerous studies have found changes in hippocampal oscillations in various neurological disorders (Tzilivaki et al., 2023; Pelkey et al., 2017). Clinical studies have established high gamma power as a potential biomarker for suicidal ideation in depressed patients (Antonoudiou et al., 2020). Our results showed that NAc-DBS recovered the CUMS-induced reduction in high gamma oscillation in the hippocampus, which may be due to neuronal recovery. We also evaluated the effect of NAc-DBS on the number of spines in the hippocampal region. Our studies revealed a significant reduction in the number of hippocampal synaptic spines in CUMS mice, which may be an important cause of cognitive impairment (Tsai et al., 2020; Guan et al., 2009). NAc-DBS treatment effectively reversed this decrease. Additionally, we detected PSD-95 expression, a critical postsynaptic scaffolding protein that regulates excitatory synapse development and plasticity (Dore et al., 2021; Levy et al., 2022). Both PSD-95 mRNA and protein expression were downregulated in CUMS mice, an effect reversed by NAc-DBS treatment.

**Figure 7:**
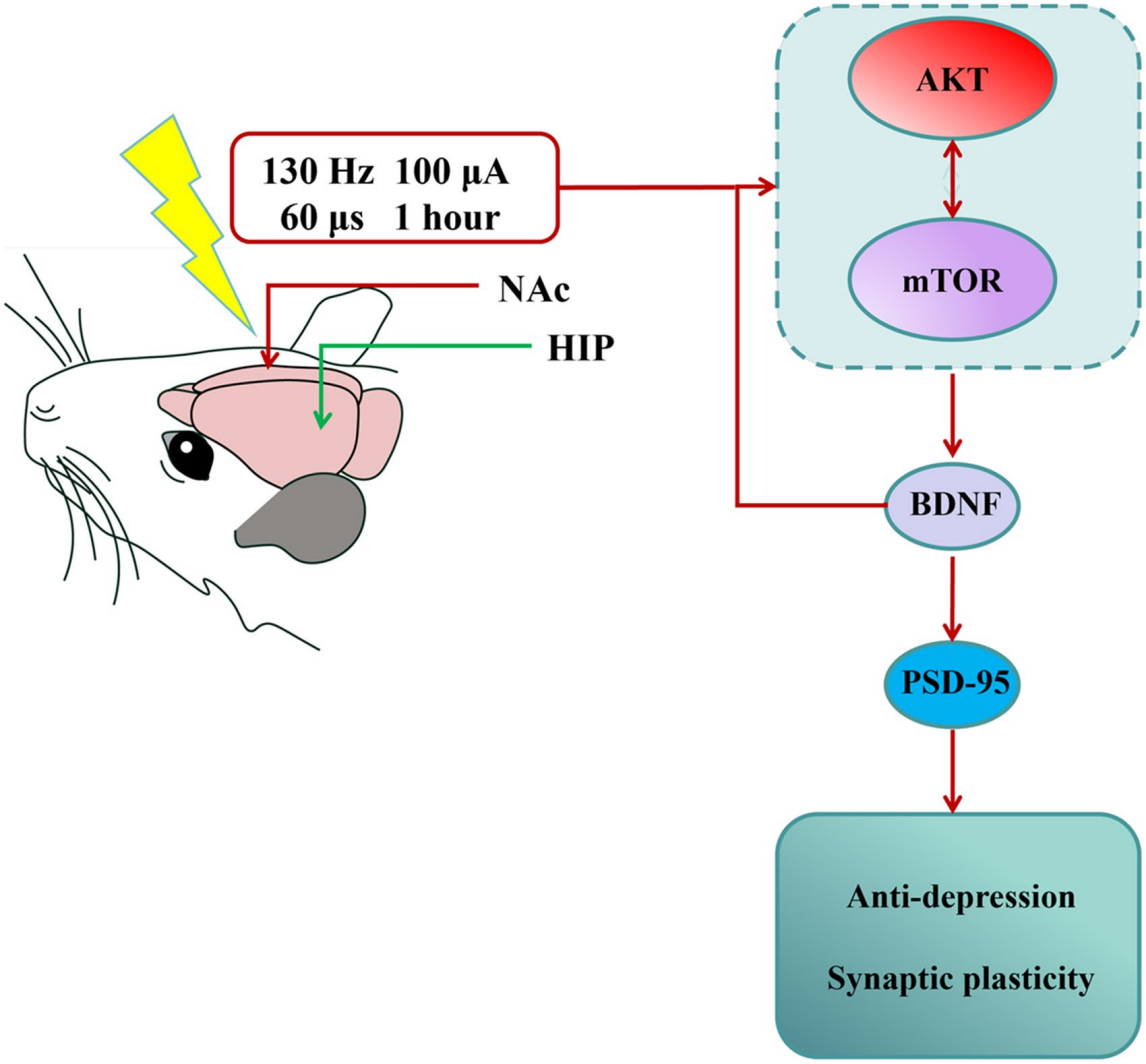
Summary of findings. NAc-DBS exerted antidepressant effect and revered synaptic plasticity in the depression-like mouse model induced by CUMS through activating AKT/mTOR/BDNF signaling pathway. The nucleus accumbens (NAc) indicated DBS location. The hippocampus (HIP) indicated LFP location.

BDNF is a ubiquitously distributed neurotrophic factor that dynamically modulates synaptic transmission and orchestrates diverse forms of synaptic plasticity (Duman et al., 2019; Wu et al., 2020). Since the formulation of the neurotrophin hypothesis of depression by Duman and Nestler in 1997, neuropathological studies consistently report decreased BDNF production in limbic–cortical circuits, with the hippocampus, prefrontal cortex, and amygdala showing particularly pronounced deficits in both human depression and experimental models (Li et al., 2023). Moreover, some antidepressants exert their effects through the BDNF signaling pathway. The PI3K/AKT/mTOR signaling cascade promotes dendritic arborization by coordinately regulating both protein synthesis and cytoskeletal reorganization (Ersahin et al., 2015; Glaviano et al., 2023; Tian et al., 2023; Su et al., 2023). As an essential downstream target for PI3K/AKT pathway, the activated mTOR pathway modulates synaptic plasticity by regulating the expression of key synaptic proteins, including PSD-95 and synaptophysin (Fakhri et al., 2021; Mayor, 2023). Interestingly, BDNF is a major activator of mTOR in neurons (Feyissa et al., 2009). Some studies also have detected that DBS and certain antidepressants that alleviate depression-like behavior may act through the mTOR signaling pathway (Jernigan et al., 2011; Liu et al., 2015). In our study, the activation of the AKT/mTOR pathway was inhibited in the hippocampus of depressive mice with reduced BDNF protein expression. However, NAc-DBS significantly reversed the levels of BDNF protein and reactivated the AKT/mTOR pathway. Furthermore, we used rapamycin, a classical mTOR inhibitor, to determine whether NAc-DBS acts through the mTOR pathway. The results showed that rapamycin aggravated depression-like behaviors that had been reversed by NAc-DBS. Interestingly, with the activation of AKT/mTOR pathway inhibited, the expression of BDNF is also reduced. Therefore, we propose that under the intervention of DBS, there may be a positive feedback regulatory loop for BDNF expression mediated by the AKT/mTOR pathway. On the one hand, this novel mechanism may partially explain why DBS can continuously and effectively improve depressive-like symptoms—the initial BDNF induced by DBS activates the AKT/mTOR pathway, and the activation of this pathway further promotes the synthesis and release of BDNF, thus forming a self-reinforcing positive cycle that continuously enhances neuroplasticity. On the other hand, this discovery also extends our traditional understanding of the interaction between BDNF and the AKT/mTOR pathway, expanding from a unidirectional “activation” relationship to a bidirectional and dynamic “mutual regulation” network, providing a new theoretical perspective for in-depth understanding of the pathophysiology and treatment strategies of emotional disorders. In conclusion, these results indicate that the antidepressant effect and cognitive improvement of NAc-DBS are related to the activation of the BDNF/AKT/mTOR signaling pathway in the mouse hippocampus. In summary, our study provides new evidence that the NAc-DBS reverses the CUMS-induced depression-like behavior and synaptic injuries in the mouse hippocampus. We also explored the antidepressant mechanism of NAc-DBS and found that it exerted antidepressant effects and cognitive improvement in a CUMS-induced depression-like mouse model through the activation of BDNF/AKT/mTOR signaling pathway. These findings provide an important theoretical basis for studying the role of NAc-DBS in the pathogenesis of depression, and will aid in identifying potential clinical intervention targets for depression (Figure 7).

However, considering the complex connections between different brain regions, further investigation is required to fully elucidate the precise neural mechanisms underlying NAc-DBS’s antidepressant effects, particularly its modulation of limbic–cortical circuits. Moreover, due to the uncertainty regarding downstream proteins of AKT/mTOR signaling pathway that play an antidepressant role in depression, further research is necessary to reveal the more precise antidepressant mechanism of NAc-DBS for its expanded clinical use. In addition, our initial study was conducted in male mice to control for variability introduced by the estrous cycle. But substantial clinical evidence indicates significant sex differences in the prevalence, symptomatology, and treatment response of depression. Consequently, our findings may not be directly generalizable to females. Our future studies are necessary to include female subjects to validate the universality of NAc-DBS efficacy and to explore potential sex-specific neural mechanisms. Furthermore, the treatment duration with NAc-DBS in this study was relatively short. Antidepressant treatments typically require several weeks to exhibit full efficacy, accompanied by persistent neuroplastic changes. Our acute or short-term stimulation protocols may not fully capture the long-lasting adaptive changes that NAc-DBS can induce, and the persistence of its effects awaits further verification. Besides, we just designed one post-treatment timepoint to determine whether NAc-DBS could relieve depression-like behavior in model mice. While, multiple post-treatment timepoints would provide valuable information on the kinetics and longevity of the effects. So that, we will design multiple post-treatment timepoints to observe the sustained effects of NAc-DBS in our further study. Finally, the potential off-target effects of rapamycin, used here as an mTOR pathway inhibitor, represent an important methodological limitation. Although rapamycin is highly selective for mTORC1 at conventional doses, higher concentrations (such as the 2 mg/kg dose used in this study) or chronic administration can affect other signaling pathways. This includes the potential for partial inhibition of mTORC2 and unintended effects mediated by interactions with other FK506-binding proteins (FKBPs). Consequently, although the behavioral rescue we observed was strongly temporally correlated with mTORC1 inhibition, we cannot entirely rule out the possibility that it was partially mediated by off-target effects. Future studies employing more precise interventional tools, such as conditional genetic knockout or ATP-competitive mTOR kinase inhibitors, are warranted to further validate the specific role of mTORC1.

## Data Availability

The raw data supporting the conclusions of this article will be made available by the authors, without undue reservation.
